# Intraperitoneal injection (IP), Intravenous injection (IV) or anal injection (AI)? Best way for mesenchymal stem cells transplantation for colitis

**DOI:** 10.1038/srep30696

**Published:** 2016-08-04

**Authors:** Min Wang, Cong Liang, Hao Hu, Lin Zhou, Bing Xu, Xin Wang, Ying Han, Yongzhan Nie, Shuyun Jia, Jie Liang, Kaichun Wu

**Affiliations:** 1 State Key Laboratory of Cancer Biology, Department of Digestive Diseases, Xijing Hospital, Fourth Military Medical University, Xi’an, 710032, China; 2 Department of Gastroenterology, Xi’an Children’s Hospital, 710006, China; 3 Department of Respiratory and Gastroenterology, Second People’s Hospital, Xi’an, 710005, China; 4 Department of Gastroenterology, PLA No.5 Hospital, Yinchuan, 750004, China

## Abstract

Stem cell transplantation showed promising results in IBD management. However, the therapeutic impacts of cell delivery route that is critical for clinical translation are currently poorly understood. Here, three different MSCs delivery routes: intraperitoneal (IP), intravenous (IV), and anal injection (AI) were compared on DSS-induced colitic mice model. The overall therapeutic factors, MSCs migration and targeting as well as local immunomodulatory cytokines and FoxP3^+^ cells infiltration were analyzed. Colitis showed varying degrees of alleviation after three ways of MSCs transplantation, and the IP injection showed the highest survival rate of 87.5% and displayed the less weight loss and quick weight gain. The fecal occult blood test on the day 3 also showed nearly complete absence of occult blood in IP group. The fluorescence imaging disclosed higher intensity of engrafted cells in inflamed colon and the corresponding mesentery lymph nodes (MLNs) in IP and AI groups than the IV group. Real time-PCR and ELISA also demonstrate lower TNF-α and higher IL-10, TSG-6 levels in IP group. The immunohistochemistry indicated higher repair proliferation (Ki-67) and more FoxP3^+^ cells accumulation of IP group. IP showed better colitis recovery and might be the optimum MSCs delivery route for the treatment of DSS-induced colitis.

Inflammatory bowel diseases (IBD) is a broad term that involves chronic inflammation of all or part of the gastrointestinal tract. Ulcerative colitis (UC) and Crohn’s disease (CD) are most common conditions in IBD. The incidence and impact of IBD is increasing worldwide. It is estimated that as many as 1.4 million Americans and 2.4 million Europeans are suffering from these diseases^1^,^2^. Furthermore, in previous low-incidents Asian area, the incidence and prevalence are also reported to increase in recent decades^3^,^4^. IBD can be painful and persistent for life, which might decrease the quality of life. For some severe cases, IBD may lead to the life-threaten complications^5^.

Therapeutic strategies should be based on a sound and thorough understanding of the disease mechanisms, if possible, however the causes of IBD are still unclear^6^. Though genetic and environmental factors are believed to be involved in the disease, not much progress has been made on therapy. Currently, therapy still largely relies on empirical, and often implemented in a stepwise fashion: progressing through 5-aminosalicylate compounds, corticosteroids, immunomodulatory drugs, and finally anti-TNF drugs^7^. These time-tested therapies may perform well on some patients, but also exhibit inadequacies in efficacy. The need of intestinal resection in CD has remained stable^8^ and the colectomy rates in UC were still for 20% and 30% within 10 and 25 years of diseases duration^9^. Thus there is a strong impetus to seek more effective approaches for disease management.

Two early reports aroused the global interests of stem cell transplantation in IBD management. Six leukemia patients with Crohn’s disease treated with allogeneic marrow transplantation, and four of five patients remained CD free 6 to 15 years after transplantation^10^. Two patients with long-standing ulcerative colitis, psoriasis, and leukemia underwent allogeneic stem cell transplantation, and all the three disorders were in clinical remission for 4 years after transplantation^11^. Since that time, the literatures using stem cells for IBD have expanded. Currently, two types of stem are used for the treatment of IBD, hematopoietic stem cells (HSCs) and mesenchymal stem cells (MSCs). From two streams of research, experimental and clinical, stem cell therapy showed promising results. However, most of these studies were focus on its therapeutic effects or mechanisms. *Very few reports pay attention to factors applied in the clinic, such as injection or exposure routes, which has highly impact on stem cell therapeutic efficiency for IBD*^12^.

In this study, three different MSCs delivery routes, intraperitoneal injection (IP), intravenous injection (IV) and anal injection (AI) were compared on DSS-induced colitis mouse model. Disease recovery was evaluated by histological server score, gross body weight and survival rate. MSCs organ distribution and engraftment was analyzed and quantified by GFP^+^ MSCs as well as Near-infrared fluorescence imaging. The levels of immunomodulatory cytokines were compared by real time-PCR and ELISA. Our findings suggested that IP delivery showed higher MSCs and better experimental colitis recovery, might be an ideal way for MSCs therapy in IBD.

## Results

### Clinical responses of DSS-induced colitis by three different MSCs delivery route

Whole marrow cells are plated in the culture dish, and non-adherent cells were removed during passage. After 3 passages, spindle-shaped cell colonies gradually predominate in the cultures. These cells were confirmed as bone marrow MSCs using flow-cytometric analysis and differential identification (Supplementary Figures S1 and S2).

**Figure 1:**
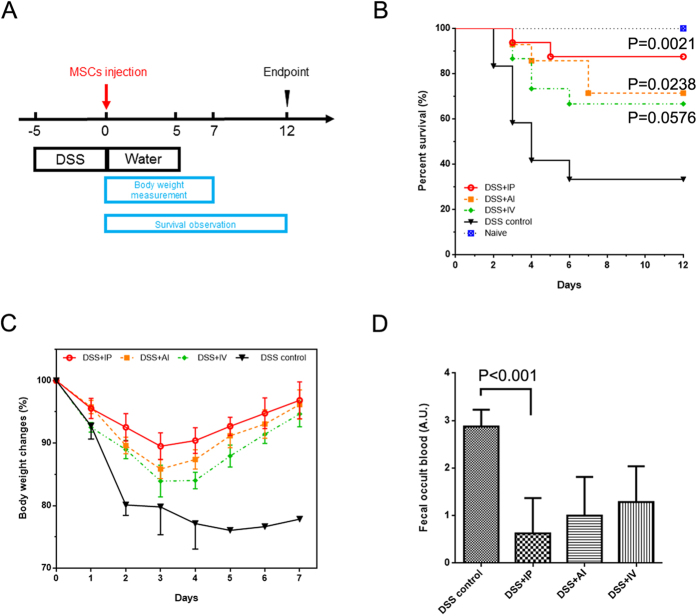
Disease recovery of DSS induced colitis after MSCs injection. (**A**) Scheme of the treatment, the day of MSCs injection was defined as day 0. (**B**) Kaplan–Meier analysis of three different MSCs delivery ways. (**C**) Percentage of body weight changes over time. (**D**) Semi-quantitative fecal occult blood test on day 3. IP, intraperitoneal injection; IV, intravenous injection; AI anal injection; n = 6–8 for treatment group, n = 3 for DSS control.

After 5 days of DSS contained water, all the subject mice showed strong positive test of fecal occult blood. These mice were then randomized into 3 groups receiving different MSCs giving therapies. The Scheme (Fig. 1A) showed the time point of DSS given and MSCs therapy. Mice were under intensive observation for 12 days. During the observation, the body weight changes and fecal blood and their survival date was faithfully record. To varying degrees, all the three MSCs delivery ways could ameliorate DSS-induced colitis. The three groups can decrease the mortality rate compared with PBS control, however, the IP injection showed the highest survival rate of 87.5% (P = 0.0021 vs. Control, Fig. 1B). Furthermore, the body weight changes displayed the less weight loss and quick weight gain of IP injection group and the maximum group difference among was reached on day 3 (Fig. 1C). The fecal occult blood test on the day 3 also showed nearly complete absence of occult blood in IP group (Figure 1D).

### IP route promotes more MSCs migration to inflamed colon

**Figure 2:**
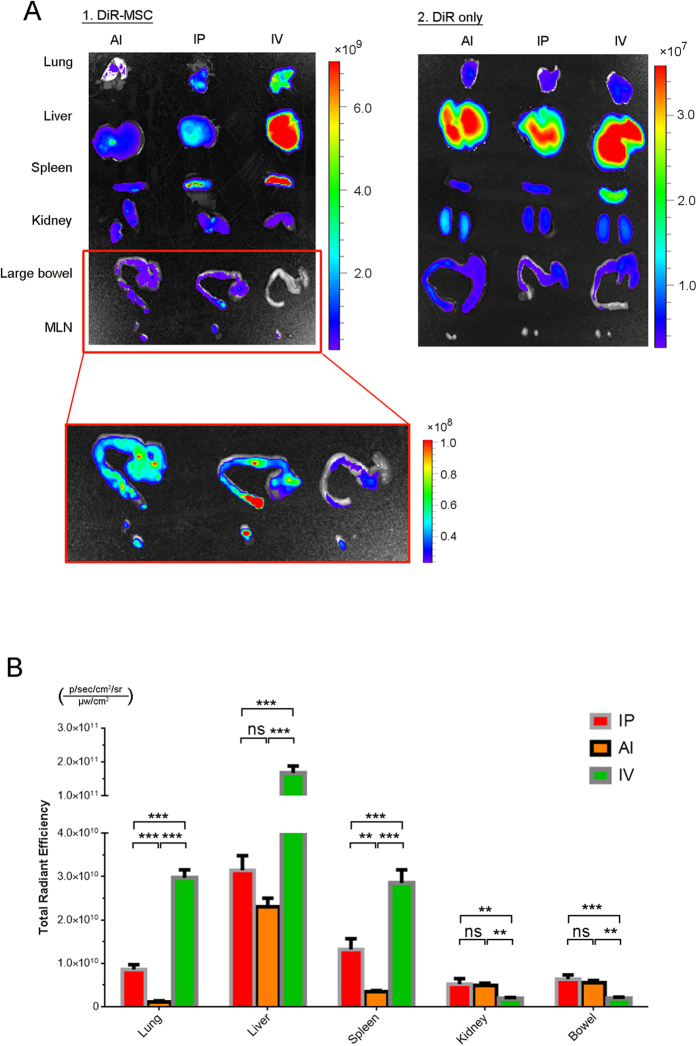
Near-infrared imaging and quantification of MSCs transplants. (**A**) MSCs were labeled with DiR dye and given by three different ways. *Ex-vivo* study was carried out at 1 day post-injection. The DiR dye alone was served as control. (**B**) MSCs signal quantification of five main organs (lung, liver, spleen, kidney, and large bowel) and corresponding MLNs. n = 3 per group. ***P < 0.001; **P < 0.01

The number of therapeutic cells that can migrate and colonize at the injury site is a decisive prerequisite for the success of cytotherapy. In order to compare the difference of MSCs colonization among the three delivery routes, a nontoxic NIR tracer DiR was introduced to label MSCs. DiR labeling showed no harm to the MSCs viability^13^ (Supplementary Figure S3). DiR-labeled cells were injected into DSS mice by three different ways. Free DiR and healthy mice (without colitis) were served as control. At 24 h after injection, the intensity of free dye was 100 folds less than the DiR labeled cells (from the bar value. 10^7 ^vs. 10^9^ ; Fig. 2A), which guarantee the less interference from the background signal that coming from free dye. While the DiR labeled cells showed very different cell distribution. In IV group, most of MSCs were trapped in the lung, liver and spleen, while the cell that immigrated to the colon was not too much. On the contrary, the IP and AI groups showed more engraftment cells at the inflamed colon but showed fewer trapped cells in lung, liver and spleen (Fig. 2A). In quantification study, the intensity of engrafted cells in IP and AI groups were significant higher than the IV (P = 0.004, P = 0.0012; Fig. 2B). Furthermore, the corresponding mesentery lymph nodes (MLNs) also showed relatively high MSCs existence (Fig. 2A). *In vivo* clearance of free DiR in healthy mice was showed in Supplemental Figure S4. Further control study comparing bowel signals on healthy mice and DSS mice after MSCs injection showed weaker NIR signal on healthy bowel in all three routes, which indicated fewer MSCs migration to the colon without colitis (Supplementary Figure S5).

**Figure 3:**
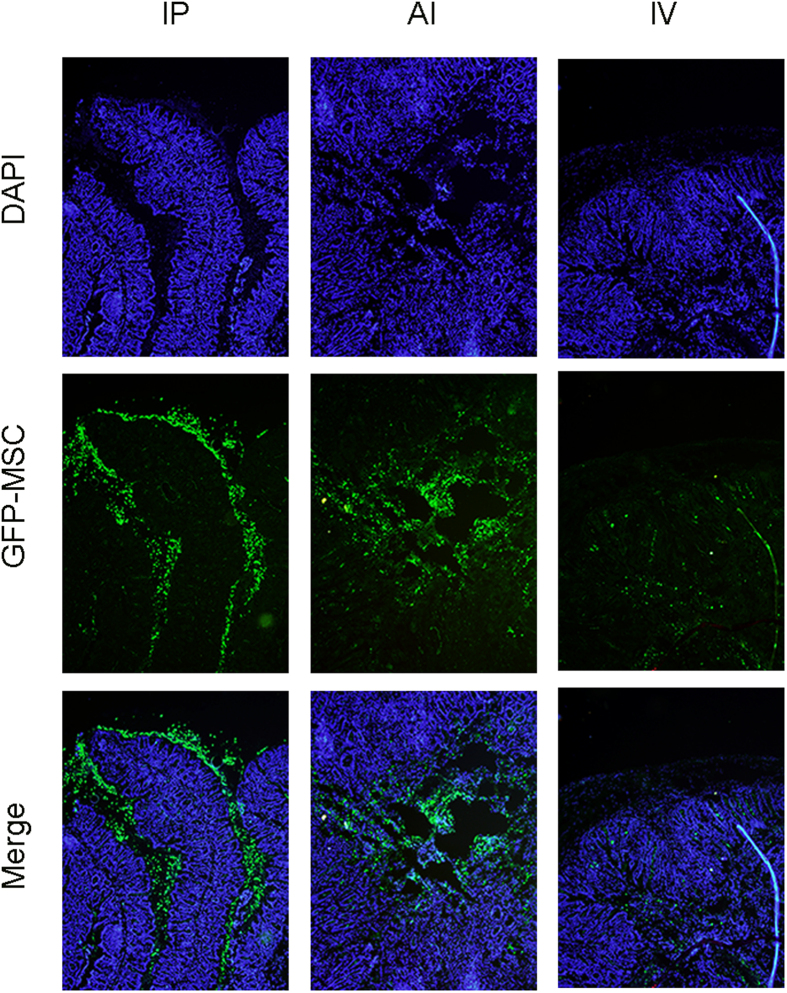
Distribution of GFP^+^ MSCs in colon 1 day after injection. GFP^+^ cells were detected at the inflamed colon. In IP group, cells were found accumulation at the epithelium; In AI group, cells were mainly in the lumen; In IV group, cells were fewer and located in lamina propria.

Meanwhile, GFP^+^ MSCs (collected from transgenic mice) were used to determine MSCs specific location. The GFP^+^ MSCs were found at inflamed colon in both IP and AI groups 24 h after MSCs injection. Interestingly, in some IP injection mice, GFP^+^ cells were observed in the epithelium of the inflamed colon. In contrast, GFP^+^ cells were showed in the lumen for AI group, and lamina propria for IV group (Fig. 3) and the IV group preserved fewer cell number. In addition, from the point of cell morphological appearance, most of MSCs kept intact in IP group, while in AI route, other than active MSCs, cell fragments and debris were also identified in the lumen (Fig. 3; Supplementary Figure S6).

### IP injection promotes better mucosal healing

**Figure 4:**
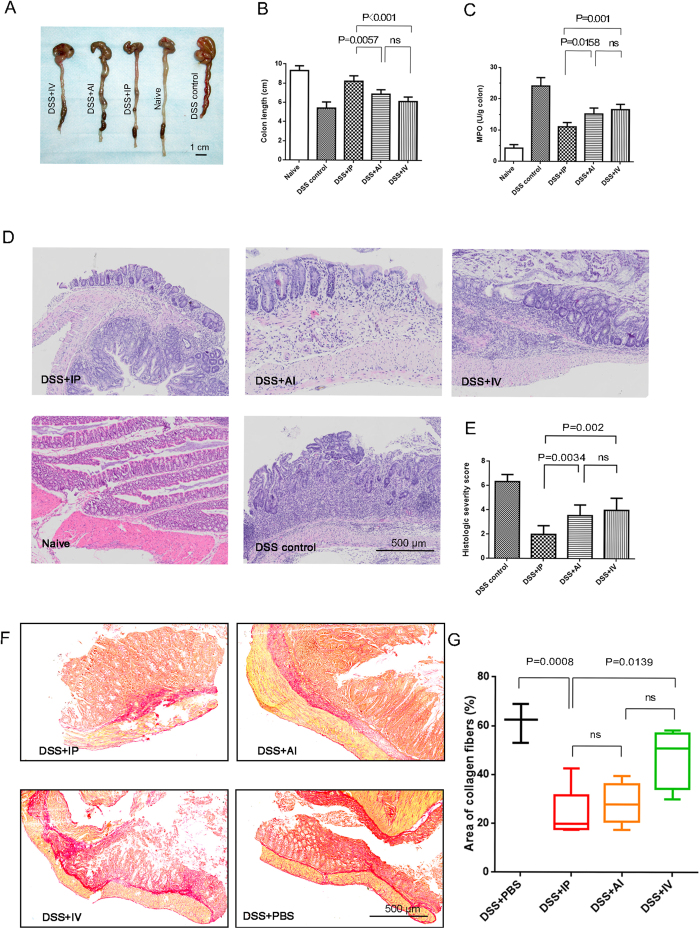
Histopathological comparison of colitis after 3 days MSCs delivery. (**A,B**) Representative colon images and quantification of colon length; (**C**) myeloperoxidase (MPO) activity in colonic protein extracts; (**D,E**) Representative H&E staining and corresponding severity score; (**F,G**) Representative sirius red staining and quantification of collagen deposition. Horizontal bars represent medians, boxes represent the 25th and 75th percentiles. n = 6–8 mice/group; n = 3 for the control and naive (health mice) group.

Since the IP injection induced more cells migration to the inflamed colon, we further compared the difference in mucosa healing of different delivery routes. We selected the day 3 as observation time point. Mice were sacrificed and colons were collected for histological analysis. From the overall appearance, the hyperemia and edema of the colon in all MSCs treatment groups became milder than the DSS control. The measurement of inflamed colon length demonstrated therapeutic effects of MSCs injection. The IP injection showed longer colon, with mean length of 8.24 cm compared with 6.90 cm in AI group (P = 0.0057) and 6.14 cm in IV group (P < 0.001; Fig. 4A,B). Other than shorten colon, colitis was also characterized by augmented colonic myeloperoxidase (MPO) activity. Reduced MPO activities were identified in all MSCs groups, of which IP injection showed lower MPO activity (P = 0.001 vs. IV and P = 0.0158 vs. AI; Fig. 4C). Severity of DSS-induced colitis was also assessed using a score system by two independent pathologists. By systemic evaluation of epithelium and lymphocyte infiltration, the IP injection group showed the relatively lower severity score compared with AI group (P = 0.034) and IV (P = 0.002), respectively. The AI and IV showed no significant intergroup difference, though AI had lower score (Fig. 4D,E).

Collagen deposition is another indicator for wound healing, however, extensive collagen deposition may hamper the gut reconstitution^14^. Sirius red staining showed disorganized collagen fibers in DSS control colon, which were diffusely distributed in the injured colon wall. With the computer-assisted software, we calculated the density of collagen fibers (%) of all groups. The IP injection significantly inhibited collagen deposition compared with DSS control (P = 0.0008) and IV group (P = 0.0139). We notice that the mean density of collagen was lowest in IP group, however, IP group did not exhibit less collagen deposition than the AI group, probably due to the scatter points (Fig. 4F,G).

### Comparison of immunomodulatory cytokine production and serum TNF–induced protein 6 (TSG-6) level

**Figure 5:**
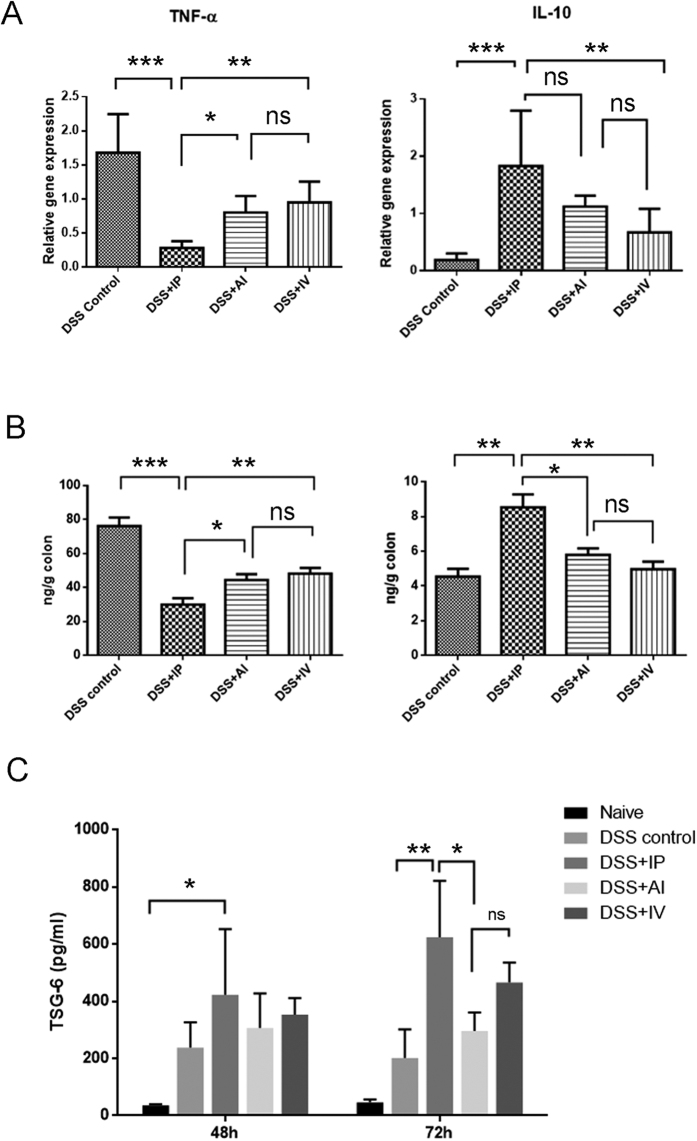
Comparison of colonic inflammatory cytokine TNF-α and IL-10 at day 3. (**A**) The colonic mRNA expression of TNF-α and IL-10 was analyzed by RT-PCR; (**B**) cytokine contents in colonic protein extracts were determined by ELISA; n = 6 mice/group; n = 3 for the control group (**C**) Serum TSG-6 level in different groups measured by ELISA. n = 3.

MSCs have been well characterized regarding their ability to produce a range of cell cytokines, which have a profound effect on modulating the immune system. Colonic mRNA expression of inflammatory mediators such as TNF-α, IL-6, IL-1β, IL-10, and IFN-γ were analyzed by real time-PCR in order to evaluate the local inflammatory environment (Fig). Generally, MSCs treatments tend to decrease the pro-inflammatory cytokine (TNF-α, IL-6, IL-1b, and IFN-γ) and increase the anti-inflammatory cytokine (IL-10). The expression level of TNF-α, the most important pro-inflammatory cytokine, was markedly decreased in IP group, compared with AI (P = 0.0174), IV (P = 0.0019) and control group (P < 0.001), respectively. On the contrary, the anti-inflammatory IL-10 level were substantially elevated in IP group compared with the IV group and DSS control, but the inter-group difference between IP and AI group, AI and IV group were not identified. For the other inflammatory cytokines, such as IL-6, IL-1β and IFN-γ, the expression levels varied among the groups and IP delivery did not show evident advantages over others (Supplementary Figure S7). In the meanwhile, the colonic homogenates were collected and colonic IL-10, TNF-α were measured by ELISA assays. In consistence with PCR results, IP groups showed higher IL-10 and lower TNF-α than other MSCs delivery ways (Fig. 5B).

We next compared serum TSG-6 level in three administration routes at 48 h and 72 h respectively after MSCs transplantation using ELISA (Fig. 5C). The data showed that the TSG-6 levels increased with time in both IP and IV injection groups, while in DSS control group, the TSG-6 level decreased from 48 h to 72 h. Although there is no significant difference among three injection groups at 48 h after MSCs transplantation, the serum TSG-6 was significant higher in IP injection group compared with naive group (P = 0.024). At 72 h, the IP groups showed significant higher level of TSG-6 than AI groups and DSS control (P = 0.023 and P = 0.005, respectively). However, no significant TSG-6 difference was found between AI and IV groups.

### Comparison of mucosa proliferation and Foxp3^+ ^infiltration

**Figure 6:**
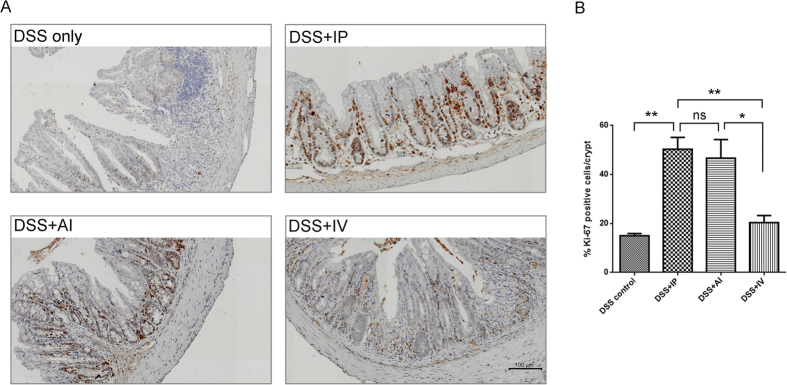
Comparison of colonic proliferation 7 days after MSCs injection. (**A**) Representative IHC images of each treatment group. MSCs induced more Ki-67 positive cells in the lower part of the crypt than the DSS control. (**B**) Quantification of % Ki-67^+^ cells/crypt, the IP and IA > IV > DSS control, n = 6 mice/group; n = 3 for the control group.

To compare the Ki-67 expression (one of the markers of cell proliferation) among the groups, the colon tissues were collected on day 7 (Fig. 6A). The Ki-67^+^ cells were greatly increased after MSCs treatment. The IP and AI groups showed mean 50.4% and 46.7% Ki-67^+^ cells/crypt respectively. In contrast, the DSS control showed 15.1% Ki-67^+^ cells/crypt. The IV group showed 20.3% ki-67^+^ cells/crypt. In addition, IP showed more intense Ki-67 stain in bottom of the crypt, which suggested more active mucosa repair proliferation (Fig. 6B).

**Figure 7:**
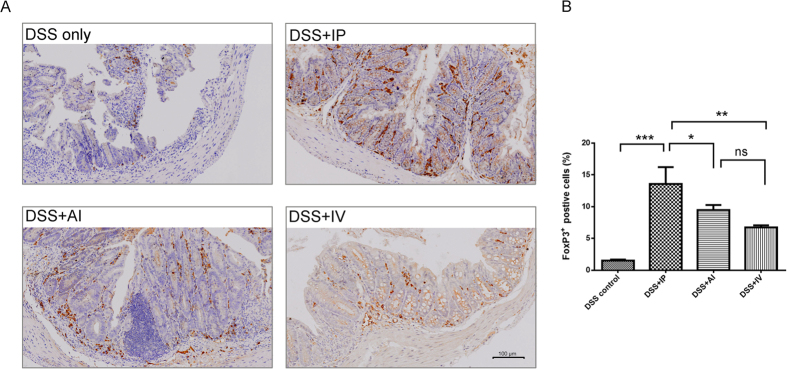
Comparison of colonic FoxP3 expression 7 days after MSCs injection. (**A**) Representative IHC images of each MSCs treatment group. FoxP3^+^ cells were greatly increased in MSCs therapy. (**B**) Quantification of FoxP3^+^ cells, the IP > IA and IV > DSS control, n = 6 mice/group; n = 3 for the control group.

There are increasing evidences showed that MSCs mediate their immunomodulatory effects through the induction of regulatory T cells (Treg cells)^14^. Here, we also compare the expression of FoxP3, which is frequently used to quantitate Treg cells. Generally, the FoxP3 expression was elevated in MSCs treatment (Fig. 7A). In DSS control FoxP3 positive cells only accounted for 1.51% of total cells, while the IP group showed 13.5% positive cells (P < 0.001). The AI groups exhibited 9.5% and IV group showed 6.7% FoxP3^+^ cells, respectively (Fig. 7B). In colonic MLNs, similar results were observed, the MSCs administration induced more FoxP3^+^ cells accumulation than the control, but the inter-group differences were not evident (Supplementary Figure S8).

## Discussion

In this study, we compared therapeutic efficacy of three MSCs administration routes in DSS-induced colitis. Our results indicated that the intraperitoneal injection is the best delivery way for MSCs, which showed better mucosa recovery and higher cell engraftment at inflamed colon. These findings may benefit clinic practice.

The optimum route of administration is an importance clinical issue not only for chemical drug, but also for stem cytotherapy. The accessibility of the therapeutic cells to their expectable target site is strongly dependent on the route of administration used. Therefore, the MSCs delivery should be tailored to the lesion kind and customized to the mechanism of action of MSCs.

Currently, the intravenous injection (IV) is historically most common methods for MSCs delivery^12^. Many published studies have showed the benefits of IV delivery in IBD treatment. However, is IV injection the best way for MSCs delivery in IBD? The first major problem with IV route is the pulmonary “first-pass” effects, which cause significant entrapment of cells^15^. This concern arises because MSCs has an estimated diameter of 20–30 μm^15^,^16^. Experiments with microspheres has showed that most of particle of this size would be clear out by the lung^17^. Certainly, cells are not microsphere which is structural rigid, they are well acknowledged about their deformation, however, a plenty of experimental data support that a substantial amount of MSCs are entrapped in the lung when following IV route^16^. Not only in animal studies, some clinical trials using MSCs to treat osteogensis imperfect (OI) in GvHD, also showed less than 1% cell were detected in target organ^18^,^19^,^20^. As expected, similar scenario was observed in our study: Compared with IP and AI, IV route indeed caused more cell entrapment in the lung, moreover, mononuclear phagocytic system, the liver and spleen also showed a great amount of MSCs present. These non-target entrapments made IV route less cells immigration and colonization, and may be a hindrance for MSCs to fully display its therapeutic effects. Second concerning is about the cell dose. Like pharmacological treatment, the cell dosing might also be an important issue for MSCs therapeutic success^21^. Increased initial cell dose ensures an increased number of cells that reach to the injury site. In most experimental studies, the MSCs treatment usually require at least 1 × 10^6^ cells/mouse, more frequently, a dose as high as 5 × 10^6^ cells/mouse to observe any effect^12^. However, for IV injection, we and others found most mice will lead increased mortality because of potential pulmonary cell embolus when the cell dose increase to 1 × 10^7^ 22. On the contrary, the most significant advantage of IP and AI routes is that cells do not immediately enter to the blood flow, allowing sufficient blood dilution. Thus, IP and AI routes are free of pulmonary embolism issue. We even tried 1 × 10^8^ cell/mouse in IP and AI group, and no immediate death was occurred. Additionally, the clinical translation is another concern. As mentioned above, a commonly used cell dose is 1 × 10^6^ cells/30 g mouse, which would be equivalent to 33 × 10^6^/kg or approximately 2.3 billion cells for a 70 kg adult. Such huge need of MSCs poses a cell source issue, which is a continuing technically and operationally challenge, since most of human ongoing clinical trials use significant less cell dose^12^. But for the IP injection, because of more cell accumulation in the inflamed colon, less initial cells might reach minimum effective cell dose. It means IP injection is more feasible and reasonable for clinical settings.

In this study, the intraperitoneal injection was demonstrated as the superior way for MSCs administration in the setting of colitis amelioration. Importantly, we identified different migration fates of MSCs and traced their ultimate location of three administration ways for the first time. This finding was consistent with previous published paper^23^, in which the intraperitoneal MSCs migrating and engraft at inflamed colon were identified by Tc-99m SPECT imaging in TNBS induced colitis. Our findings showed that the GFP^+^ MSCs were migrated to the inflamed colon, even passed through the whole intestinal wall and reached the luminal side. This is really an interesting finding, however exact mechanisms are still unknown. Chemotactic cytokines might be involved and responsible for MSCs attraction. Study showed that genetically modified MSCs to increase CXCR-4 expression will lead to an increase of MSCs migration to intestinal with radiation enteritis and then resulted in disease improvement^24^. Another study by coating MSCs with VCAM-1 also exhibited an increased MSCs migration to the inflamed colon and promoted tissue repair capacity^25^. In our study, we found inflamed colon could induce more MSCs engraftment than heathy colon, which partly supported this hypothesis, since inflamed colon has been reported of releasing variety of chemotactic cytokines^26^.

But how these miracle cells reach to the lumen and by which possible migration route? Taede *et al*. study on peritoneal cells (PCs) migration to gut may give us some hints^27^. In their study, PKH26 (a yellow-orange fluorescent dye) labeled PCs were injected intraperitoneally into healthy rat. They found that PCs can directly migrated from peritoneal cavity and some of them were found at intestinal villi. Because labeled cells are founded in Peyer’s patch of the gut and peripheral blood, they proposed the most likely routes might be by both lymph- and blood circulation. However, they found the migrated PCs was very few, and these may attribute to: 1) the recipient are heathy rat. Gut epithelium is intact and lacks cytokines or chemokines attraction; 2) most of PCs are immune cells, such as B cells (50–60%), macrophages and mast cells (30%) and T cells (5–10%)^28^. Unlike MSCs, they are restricted by migration because of receptors in the peritoneal cavity specially recognizing the PCs^29^,^30^. But this study at least pointed out the possible migration route for cells by intraperitoneal injection.

However, one recent published paper showed inconsistent results with ours, in which IV injection was identified better in comparison with intraperitoneal injection^31^. This inconsistency may attributed to different MSCs types. Our work used marrow-derived MSCs but not adipose-derived ones. These two kinds of MSCs differ in proliferation rates and differentiation capacities as well as show significant difference in cytokine secretome and chemokine receptor expression which may affect migration, engraft and even local function^32^,^33^,^34^,^35^,^36^. Indeed, Mariana *et al*. evaluated the therapeutic efficacy of different MSCs sources and delivery routes in experimental emphysema^37^. They found different source of MSCs with different delivery routes exhibited distinct effects on lung and cardiovascular injury. Therefore, the biological differences of different source MSCs should be considered systematically when interpreting the research results and choosing for specific clinical application.

The MSCs may exert their therapeutic effects on mainly three following aspects: firstly, to differentiate and replace the damage tissue; secondly, to secrete soluble immunomodulatory bioactive molecules and establish a regenerative milieu; thirdly, cell-to-cell contact and interaction with immune cells.

MSCs has been found to directly differentiate into end-stage cells to augment bone damages and repair cartilage defects^38^,^39^. Some research showed exogenous CD34 stem cells migration towards the inflamed colon and differentiation into endothelial cells^40^. However, more studies described MSCs as “hit and run” therapy, which discovered most of MSCs died and cleared from the body within 48 to 72 h^20^,^41^,^42^,^43^. In our study, we tracked the GFP^+^ MSCs colon colonization, but our results also showed the short stay of MSCs after injection. In IP group, the MSCs existence can be detected at day 1, but gradually decreased with the time, GFP^+^ cells can be hardly identified in inflamed colon after day 3 (data not shown). In IV group, the colon homing cells were fewer, most of MSCs were trapped in MSCs were rapidly accumulate into the lung and mononuclear phagocytic system. Admittedly, there may be an overlook of MSCs existence due to the tissue selection of frozen section and sensitivity of fluorescence imaging, the therapeutic differentiation of MSCs in our study were not clearly observed. And the loss of MSCs in the colon may attribute to many reasons, such as, wash out, cell death, or even rejection via the innate immune system.

MSCs has been well characterized of producing a range of modulatory cytokine, which inspires the designation of these cells as “injury drugstore”^44^. Other than preformed at the site of injury, MSCs has showed ability to exert their therapeutic effects distally. In study by Pennesi *et al*., MSCs was found to be able to prevent damages caused by collagen-induced arthritis, despite of lack of a detectable presence in the arthritic joint^45^. Lee *et al*. found MSCs improved cardiac function by a remote way^46^. Most recently published study by Scaldaferri *et al*. seem to have found “the key drug” of MSCs in the treatment of colitis, TSG-6^47^. Their findings showed that intraperitoneally injected MSCs would form aggregates with macrophages and lymphocytes in peritoneal cavity and remotely secreted TSG-6. Further study demonstrated TSG-6 alone was sufficient to reduce intestinal inflammation in mice with colitis. TSG-6 might be a major anti-inflammatory mechanism of MSCs, since not only in colitis model, the therapeutic effect of TSG-6 remotely secreted by MSCs were also identified in many other disease model^46^,^48^,^49^,^50^. We also evaluated serum TSG-6 in three MSCs administration route. In consistent with Scaldaferri’s work, the increased TSG-6 after MSCs transplantation was also identified. Most importantly, IP was found to have highest serum TSG-6 at both 48 h and 72 h, and showed statistical significance when comparing with AI and DSS control at 72 h. These findings, to some extent, may help to understand why IP is better than AI and IV. Firstly, peritoneal cavity provides an isolated, hemodynamically stable, sterile and nutritious environment with enough growth spaces, which are very critical factors for aggregates forming. In contrast, MSCs were either infused with the bowel waste (for AI route) or trapped in lung/liver vessels (for IV route)^51^, where aggregates forming would be affected by unfavorable conditions, such as contaminated microenviroment, limited growth space and hemodynamically unstable in lung/liver vessels. In addition, even MSCs could formed aggregates in those harsh conditions, there still be a highly risk of self-removal. For example, discharged accompany with stool or cleared out by lung microphage. Secondly, as mentioned above, there are plenty of immune cells in peritoneal cavity^28^, which are indispensable component of MSCs aggregates. These immune cells may interact with MSCs and exert regulatory functions to MSCs. For this reason, more MSCs aggregates would form for IP route and produce higher level of serum TSG-6 under TNF-α activation, which help reducing the colon inflammation.

The increasing evident indicated that cell-cell contact between MSCs and immune cells may also be of importance^52^,^53^,^54^. Just mentioned above, immune cells and MSCs interaction in aggregates promotes TSG-6 secretion, and contributed to the colitis recovery. In addition, MSCs showed inhibitory effects on proliferation of memory T-cells by direct cell contact^52^. MSCs also showed ability to promote epithelial repair^54^ and regulate dendritic cells by cell-cell contact mechanism^55^,^56^. In our study, we also identify MSCs existence in nearby MLN. The good cell-cell cross talk between MSCs and immune cells may be another contributor for better therapeutic effects.

Though IP injection showed benefits in many human and animal studies, even recommend by National Cancer Institute (NCI) for advanced ovarian chemotherapy, it is more often applied to animals than to humans for the time being^57^. The complications associated with IP injection are major concern. These complications include catheter infection, physical damages to intra-abdominal structures, such as vaginal vault perforation, bladder erosion, and bowel perforation^58^, and direct drug toxicity to non-specific abdomen organ (e.g. some potent chemo and radio-labeled drugs)^59^. Researchers has tried many creative ways to minimize these risks. One example is using ultrasound-navigation for puncture. It can significant reduce failure puncture and associated organ damages caused by blind needle puncturing. Another example is applying “hammock” catheter placement technique^60^. By using this techniques, the catheter complications, in particular obstruction, bowel and vaginal cuff perforation were greatly reduced^61^. We believe with the increasing interests for IP injection in the field of cytotherapy, more innovation and creative ideas for reducing IP complications will come up.

## Conclusion

In this study, the therapeutic efficiency of three different MSCs delivery routes (IP, AI and IV) were compared on DSS-induced colitis mouse model. From whole-body disease recovery, the amount of MSCs colon’s engraftment, tissue histological evaluation as well as levels of cytokines and TSG-6. IP injection showed better amelioration of colitis and may be an ideal delivery way for MSCs in IBD therapy.

## Methods

### Animals

C57BL/6 mice were brought from (6–8 weeks; SLACCAS, Shanghai, China), GFP transgenic mice ware brought from (The institute of Laboratory Animal Science, Chinese Academy of Medical Sciences & Peking Union Medical College, China). Mice were maintained in a light/temperature-controlled room and allowed to freely access to chow diet and water. All experimental procedures performed were approved by Animal Welfare and Ethics Committee of the Fourth Military Medical University (FMMU), which are in accordance with the NIH guidelines.

### MSCs Isolation and Culture

MSCs were isolated from C57BL/6 mice bone marrow as previously reported^62^. Generally, the mice were killed by cervical dislocation. The bilateral femurs and tibias were aseptically excised, stripped of connective tissues, and then stored in PBS. Then, the bone marrow was flushed out with 1 mL syringe. After centrifuge, the cells were certificated and transferred to MSC expansion culture medium consisting of a-minimum essential medium (a-MEM; Gibco, USA) supplemented with 10% fetal bovine serum (FBS; Gibco, USA) and 100 μg/mL penicillin/streptomycin. The medium was refreshed every 2–3 days and third- and fourth-passage cells were used for all experiments.

### Acute Colitis Induction and Cell Transplantation

Acute colitis was induced in C57BL/6 male mice by feeding 5% DSS (molecular weight 40000 Da; Sigma) dissolved water for 5 days, followed by five days of regular drinking water according to our previously published study^63^. At day 5, mice were randomized and injected with MSCs (1 × 10^6^, 200 μL in volume) by three delivery routes (IP, AI and IV). DSS only served as control. In anal injection group, same amount of cells were suspended in 200 μL diluted Matrigel in PBS (1:20), and the cell suspension was instilled into colonic lumen by a syringe with flexible catheter 4 cm in length and 2 mm in diameter, according to the previous study^64^.

### NIR dye labeling and Fluorescent Imaging

NIR fluorescent dye, 1, 1′-dioctadecyl-3,3,3,3′-tetramethylindotricarbocyanine iodide (DiR; DiIC18(7), Molecular Probes, Invitrogen, Carlsbad, CA) was used for labeling the cells as previously reported^65^. The excitation/ emission spectrum of DiR is in the near infrared range (excitation 750 nm and emission 782 nm). Every 1 × 10^7^ cells were incubated with 10 mL DiR solution (PBS based containing 3.5 μg/mL dye and 0.5% ethanol) for 30 min at 37 °C. The labeled cells were twice with warm fresh medium at 1500 rpm for 5 min to ensure complete removal of any unbound dye. All fluorescent imaging procedures were conducted on small animal imaging system (IVIS Kinetics; Caliper Life Science). Fluorescent intensity was quantified and processed using the Living Image Software (Version 4.2; Caliper Life Science).

### Histological Inflammatory Scores and Collagen Deposition

Three days after MSCs transplatation, the mice were sacrificed for histological evaluation (H&E and phosphomolibidic acid-picrosirius red staining). Histological evaluation was completed in a double-blind fashion by two independent pathologist. Inflammatory Scores were calculated using a score system^66^: Epithelium (E): 0, normal morphology; 1, loss of goblet cells; 2, loss of goblet cells in large areas; 3, loss of crypts; 4, loss of crypts in large areas. Infiltration (I): 0, no infiltrate; 1, infiltrate around crypt basis; 2, infiltrate reaching to lamina muscularis mucosa layer; 3, extensive infiltration reaching the muscularis mucosa with abundant edema; 4, infiltration of the submucosa layer. The histological score was defined as the sum of the two parameters (total score = E + I). Collagen Deposition was assessed with phosphomolibidic acid-picrosirius red staining. Quantification of collagen fiber was assessed using Image Pro Plus software (version 6.0).

### Test for Fecal Occult Blood

Fresh feces from animals were collected for occult blood test, using fecal occult blood kit (Baso Diagnostics Inc., Zhuhai, China) according to the manufacture’s recommendations. Tests were read by an independent observer (Cong Liang) and scored 0–5 as shown by the color indicators provided by the manufacturer.

### Myeloperoxidase (MPO) activity assay

Neutrophil infiltration was monitored by measuring MPO activity^67^. Briefly, colonic segments were homogenized at 50 mg/mL in phosphate buffer (50 mM, pH 6.0) with 0.5% hexadecyltrimethylammonium bromide. Samples were frozen and thawed 3 times, centrifuged at 30,000 g for 20 minutes. The supernatants were diluted 1:30 with assay buffer consisting in 50 mM phosphate buffer pH 6.0 with 0.167 mg/mL o-dianisidine (Sigma) and 0.0005% H_2_O_2_.The colorimetric reaction was measured at 450 nm between 1 and 3 min in a spectrophotometer (Varioskan™ Flash Multimode Reader, Waltham, MA). MPO activity per gram of wet tissue was calculated as: MPO activity (U/g wet tissue) = (A_450_) (13.5)/tissue weight (g). The coefficient 13.5 was empirically determined such that 1 U MPO activity represents the amount of enzyme that will reduce 1 μmol peroxide/min.

### RNA extraction and quantitative real time PCR

mRNA expression of inflammatory mediators such as TNF-α, IL-6, IL-1b, IL-10 and IFN-γ in the colon were measured by real time PCR. Total RNA was extracted with the RNAeasy Plus kit (QIAGEN). RNA was reverse transcribed using the iScript cDNA Synthesis Kit (Bio Rad, Hercules, CA). iQ SYBR Green Supermix and CFX96 (Bio-Rad) were used for template amplification with a primer for each of the transcripts examined. All reactions were performed in triplicated. GAPDH mRNA was used as an internal control to normalize mRNA expression. The primer sequences used were listed in Supplementary Table S1.

### Inflammatory cytokine and Serum level of TSG-6 analysis by ELISA

To determined cytokine in colon mucosa, protein extracts were isolated by homogenization of colonic segments (50 mg tissue/mL) in 50 mmol/L Tris-HCl, pH 7.4, with 0.5 mmol/L dithiothreitol and 10 μg/mL of a cocktail of proteinase inhibitors (Sigma). Samples were centrifuged at 30,000 g for 20 minutes and stored at −80 °C until cytokine determination. Cytokine in colonic protein extracts were determined by ELISA Kits according to the manufacturer’s instructions (R&D Systems, Minneapolis, MN). The serum concentration of TNF–induced protein 6 (TSG-6) was measured in DSS colitic mice at 48 and 72 hours after different routes of MSCs administrations. The ELISA kit for TNFα–induced protein 6 was used (CUSABIO Life science, China). Samples were prepared and tested as previously reported^47^.

### Immunochemistry staining (Ki67 FOXP3)

IHC was performed according to the standard procedures. In brief, sections were dewaxed and rehydrated by xylol and acohol. After antigen retrieval, the endogenous peroxidase activity was blocked by 1% H_2_O_2_ for 10 min. Then sections were washed and blocked with normal nonimmunone goat serum for 30 min. Afterwards, the samples were incubated with Ki-67 (1:200; Santa Cruz) or FOXP3 (1:100; Antibodies-Online, Aachen, Germany) overnight at 4 °C. After wishing with PBS, the sections were incubated 1 h at room temperature with secondary antibody, then visualized by diaminobenzidine (DAB). Normal mouse IgG was used as a substitute for the primary antibody as negative control. Proliferation index was defined as percent of Ki-67 positive nuclei within the random visual scope. All the tissue sections in this study were scan and analyzed with Virtual slide scanning system (VS120-S, Olympus).

### Statistical analysis

Quantitative data are presented as the mean ± SD. Statistical significance was determined using one-way ANOVAs or Student’s t tests. P values of <0.05 were considered statistically significant. Survival curve plots and Kaplan-Meier analyses were performed using Prism 5.0 software.

## Additional Information

**How to cite this article**: Wang, M. *et al*. Intraperitoneal injection (IP), Intravenous injection (IV) or anal injection (AI)? Best way for mesenchymal stem cells transplantation for colitis. *Sci. Rep.* **6**, 30696; doi: 10.1038/srep30696 (2016).

## Supplementary Material

Supplementary Information
