# Supplementary material for: Intraperitoneal injection (IP), Intravenous injection (IV) or anal injection (AI)? Best way for mesenchymal stem cells transplantation for colitis
Source: Sci Rep. 2016 Aug 4;6:30696. doi: 10.1038/srep30696 (PMC4973258; doi:10.1038/srep30696)
Supplement: Supplementary Information [file srep30696-s1.pdf]

Intraperitoneal injection (IP), Intravenous injection (IV) or anal injection (AI)? Best way for mesenchymal stem cells transplantation for colitis

Min Wang, Cong Liang, Hao Hu, Lin Zhou,

Bing Xu, Xin Wang, Ying Han, Yongzhan Nie, Shuyun Jia,

Jie Liang, Kaichun Wu

Supplementary methods

Characterization of MSCs

Flow cytometric analysis was used for the characterization of surface protein expression of MSCs. Cells of passage 3-4 are harvested by trypsin digestion and stained for phycoerythrin (PE) conjugated anti-moue CD73, CD90, CD105, CD34 or fluorescein isothiocyanate (FITC) conjugated anti-mouse CD45 and CD11b antibody. All the antibodies and their isotype wereobtain from BD Bioscience.

MSCs differentiation assay

Osteoblast, adipocyte and chondrogenic differentiation were initiated by means of specific media. For osteogenic differentiation, the medium contained 0.01 μM dexamethasone, 10 mM β-glycerophosphate, and 5 μM ascorbic acid, while for adipogenic differentiation, medium contained 1 μM dexamethasone, 500 μM isobutylmethylxanthine, 100 μM indomethacin and insulin 10 ug/mL (all from Sigma Aldrich, St Louis, MO). Intracellular lipid or Calcium deposits can be stained with Oil Red O or Alizarin Red S method. For chondrogenic differentiation, differentiation medium was prepared in DMEM, supplemented with 6.25 μg/mL insulin, 5 μg/mL ascorbic acid 2-phosphate and 10 ng/mL TGF-β (Weikai Bioeng Co. Ltd, Tianjin，China). Proteoglycans presence was verified by toluidine blue after 21 d of induction.

In vivo clearance of DiR in healthy mice

10 mL DiR cell labeling solution (PBS based containing 3.5 μg/mL dye and 0.5% ethanol) were prepared as Methods. 200 μL of labeling solution was injected into BALB/c nude mice by three delivery routes (IP, AI and IV). The whole-body *in vivo* imaging was conducted at 10 min, 1 h, 2 h, 6 h and 24 h after injection by small animal imaging system with same settings (IVIS Kinetics; Caliper Life Science). After imaging, the mice were sacrificed. Main organs (heart, liver, spleen, kidney, and bowel) and blood were harvested and imaged, respectively.

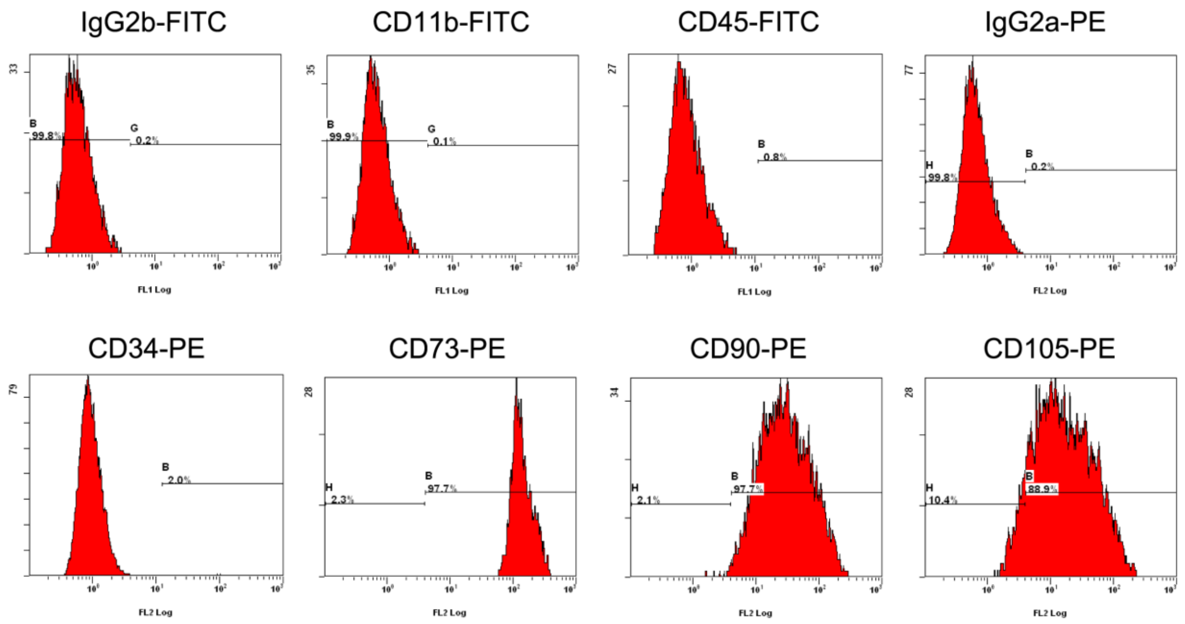

Supplementary Fig S1. Flow cytometry analysis for the expression of cell surface antigens of MSCs.

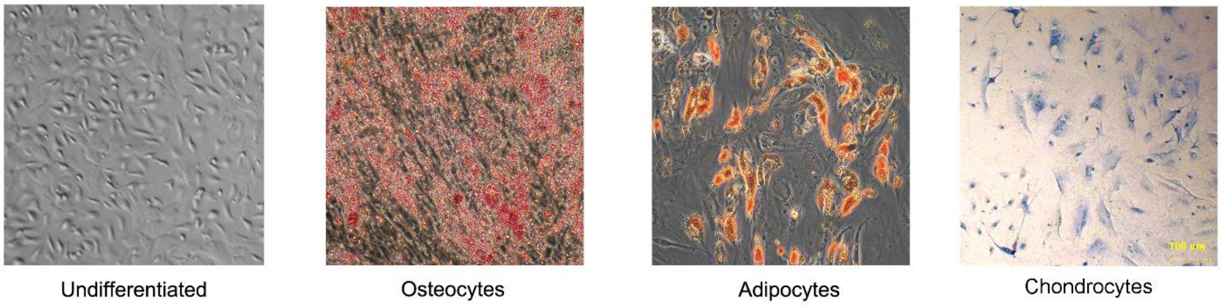

Supplementary Fig S2. Cell morphological appearance of undifferentiated MSCs and MSCs differentiated into adipocytes, osteocytes and chondrocytes.

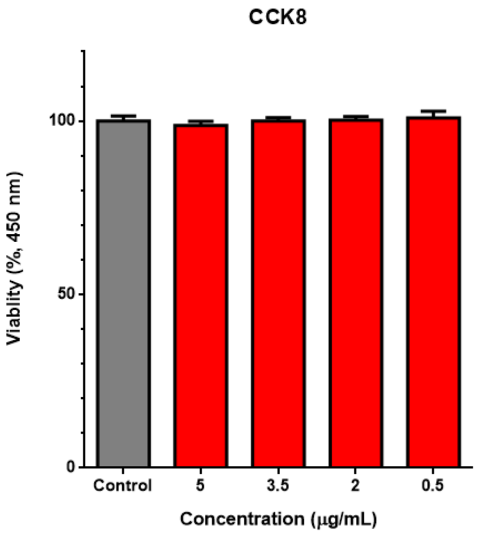

Supplementary Fig S3. CCK8 viability analysis of MSCs after different concentration of 1, 1'-dioctadecyl-3,3,3,3'-tetramethylindotricarbocyanine iodide (DiR; DiIC18(7) labeling.

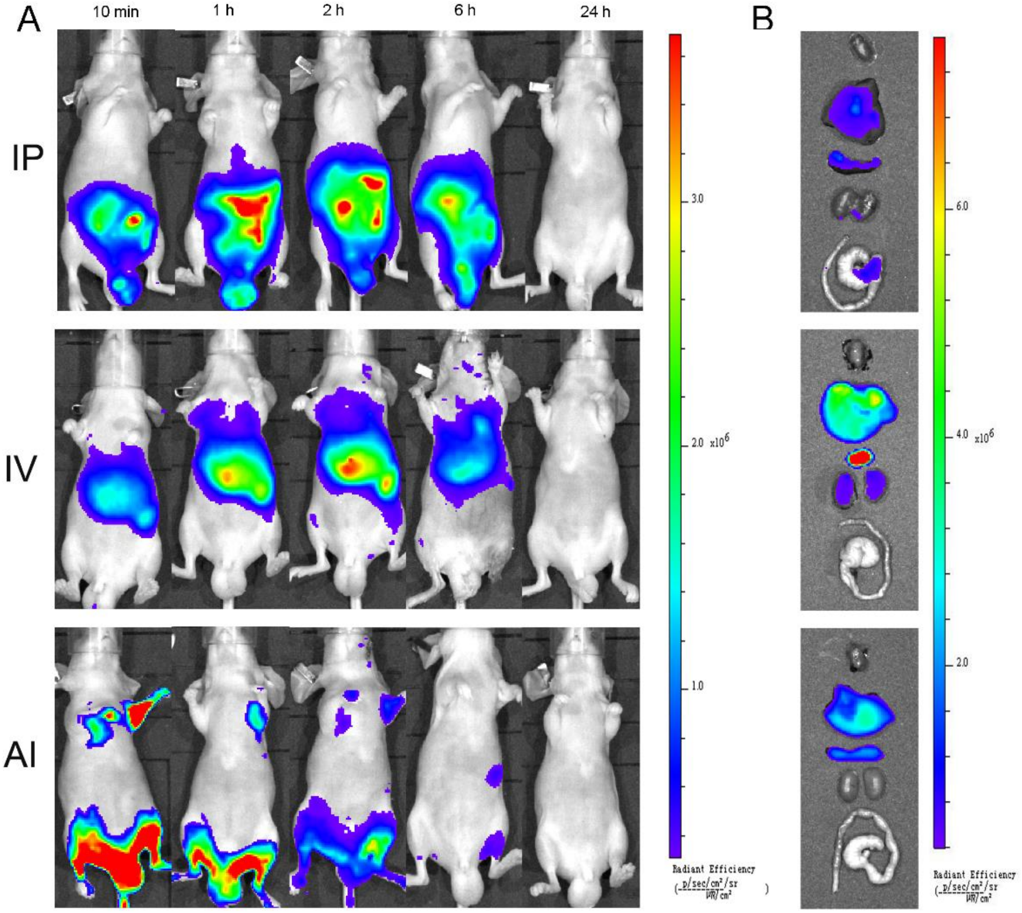

Supplementary Fig S4. *In vivo* clearance of free DiR dye in healthy BALB/c nude mice, Imaging was taken at 10 min, 1 h, 2 h, 6 h and 24 h post injection. Free DiR showed quick clearance in body. At 24 h, most of Dye was cleared from body. (A) Upper panel: IP route; Middle panel: IV route; lower panel AI, route (n=3). (B) Representative images of DiR organ distribution in three different routes at 24 h post injection (From top: heart, liver, spleen, kidneys and bowel).

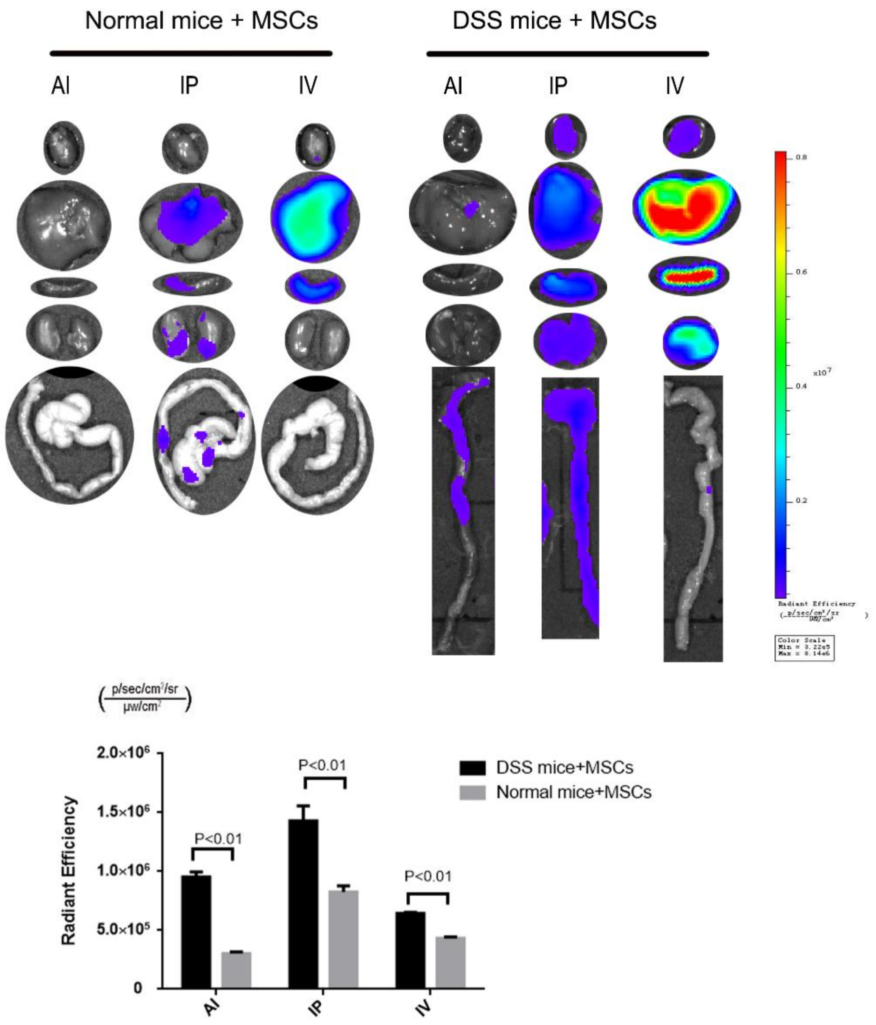

Supplementary Fig S5. MSCs distribution in healthy and DSS colitis mice after three different administration routes. (A)Upper panel: representative images of MSCs in 5 main organ after 24 h injection (From top: heart liver, spleen, kidney and bowel); (B) Comparison of fluorescent intensity in bowel of healthy and DSS mice (n=3).

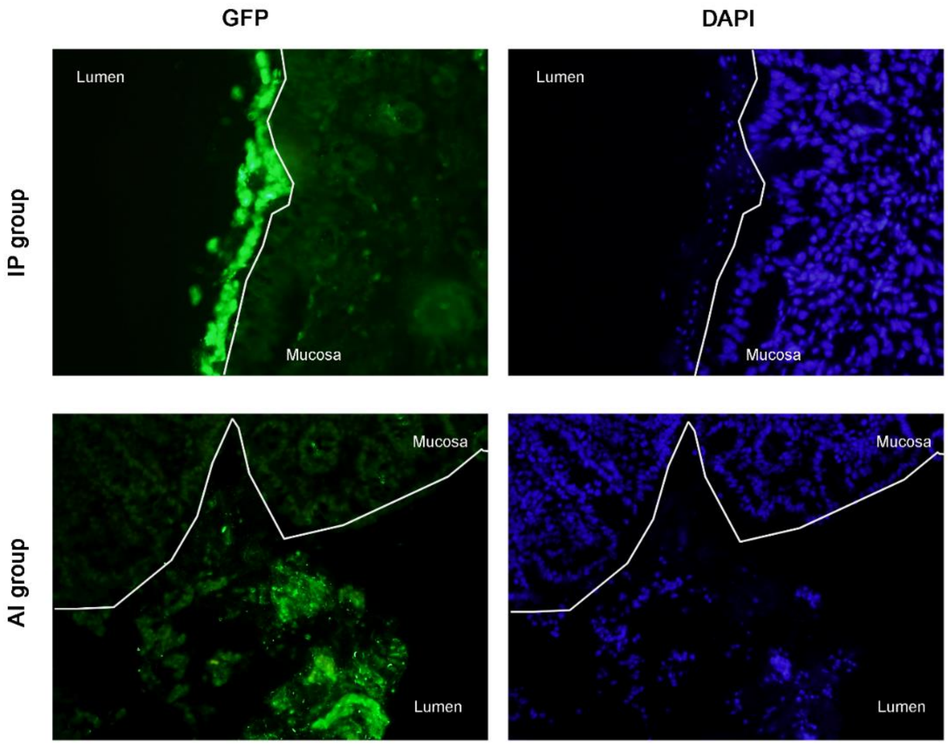

Supplementary Fig S6. Morphological appearance of GFP^{+} cells in IP and AI group at inflamed colon (magnification ×200).

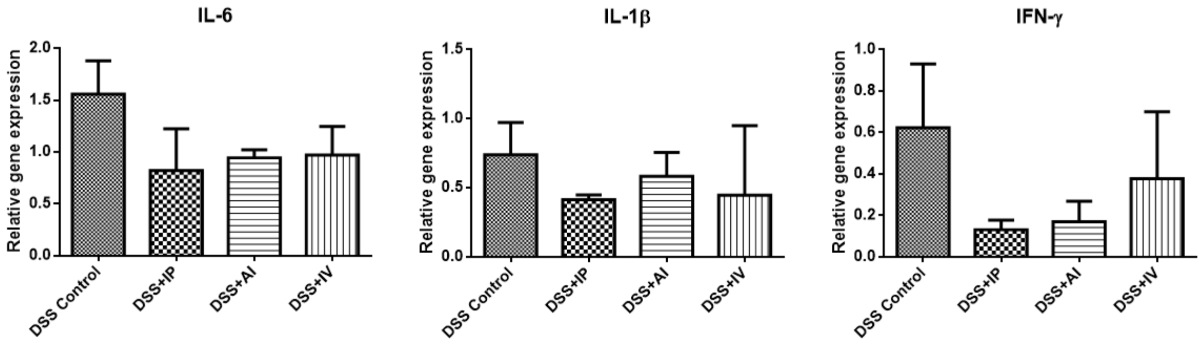

Supplementary Fig S7. The colonic mRNA expression of IL-6, IL-1β and IFN-γ analyzed by RT-PCR.

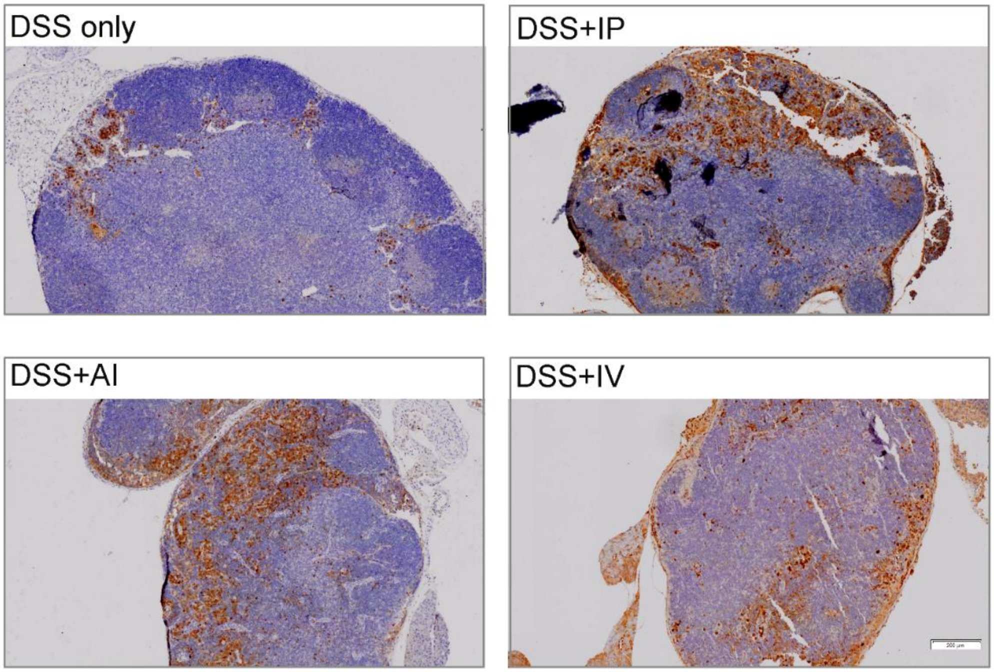

Supplementary Fig S8. FoxP3 expression in MLNs. MSCs administration induced more FoxP3$^+$ cells accumulation than the control, but the inter-group differences were not evident.

Supplementary Table S1 List of primers for real time PCR

GENE	Forward primer (5'-3')	Reverse primer (5'-3')
**TNFα**	TATGGCCCAGACCCTCACA	GGAGTAGACAAGGTACAACCCATC
**IL-1β**	TCCAGGATGAGGACATGAGCAC	GAACGTCACACACCAGCAGGTTA
**IL-10**	GCCAGAGCCACATGCTCCTA	GATAAGGCTTGGCAACCCAAGTAA
**IL-6**	CCACTTCACAAGTCGGAGGCTTA	CCAGTTTGGTAGCATCCATCATTTC
**IFN-γ**	CGGCACAGTCATTGAAAGCCTA	GTTGCTGATGGCCTGATTGTC
**GAPDH**	TGTGTCCGTCGTGGATCTGA	TTGCTGTTGAAGTCGCAGGAG
